# Context, cortex, and associations: a connectionist developmental approach to verbal analogies

**DOI:** 10.3389/fpsyg.2013.00857

**Published:** 2013-11-20

**Authors:** Pavlos Kollias, James L. McClelland

**Affiliations:** ^1^Department of Psychology, Princeton UniversityPrinceton, NJ, USA; ^2^Department of Psychology, Center for Mind, Brain and Computation, Stanford UniversityStanford, CA, USA

**Keywords:** analogical reasoning, connectionist models, cognitive development, FTLD, cognitive control, word association

## Abstract

We present a PDP model of binary choice verbal analogy problems (A:B as C:[D1|D2], where D1 and D2 represent choice alternatives). We train a recurrent neural network in item-relation-item triples and use this network to test performance on analogy questions. Without training on analogy problems *per se*, the model explains the developmental shift from associative to relational responding as an emergent consequence of learning upon the environment's statistics. Such learning allows gradual, item-specific acquisition of relational knowledge to overcome the influence of unbalanced association frequency, accounting for association effects of analogical reasoning seen in cognitive development. The network also captures the overall degradation in performance after anterior temporal damage by deleting a fraction of learned connections, while capturing the return of associative dominance after frontal damage by treating frontal structures as necessary for maintaining activation of A and B while seeking a relation between C and D. While our theory is still far from being complete it provides a unified explanation of findings that need to be considered together in any integrated account of analogical reasoning.

## 1. Introduction

Analogical reasoning, the ability to detect and exploit patterns of relational similarity between domains of knowledge, has been argued to be at the core of human cognition (Hofstadter, 2001). Studies and models have focused on different aspects of analogical reasoning. According to the number of constituents that the two knowledge domains will have, the form of the questions that the task will assume, and other variables, different paradigms have been developed. Some studies have focused on the processing of analogous domains of knowledge and situations where many objects are related with each other (Duncker, 1945; Gick and Holyoak, 1983). In this case, the entities in the knowledge domains are assumed to have a form of structure that can be mapped with entities in an analogous domain as a result of analogical reasoning (Gentner, 1983). In some studies, the subjects are explicitly asked to solve an analogy problem, while in others their capability to spontaneously infer an analogy is tested, mainly for goal-directed problem solving tasks (Duncker, 1945; Gick and Holyoak, 1983; Holyoak et al., 1984).

In one important type of explicit analogy problems, participants see three items (A:B::C) and must select a fourth item to complete an analogy of the form “A is to B as C is to D” (Spearman, 1923; Sternberg and Nigro, 1980; Sternberg et al., 1982). Participants, commonly, are given a set of candidate D items and must choose the option that maximizes the similarity of the relation between A and B with the relation between C and the picked D. Such forced-choice verbal analogy problems are often used in standardized tests of mental ability, and researchers have examined performance of adults and children either using pictorial presentation of objects and scenes (Goswami and Brown, 1990; Kotovsky and Gentner, 1996) or verbally (Gentile et al., 1969, 1977; Sternberg and Nigro, 1980).

In this paper we seek to provide an integrated account of both developmental and neuropsychological findings from studies employing forced choice verbal analogy problems. The number of candidate options for the D item is not constant across studies. Depending on the variables of interest, studies have used, two (Morrison et al., 2004), four (Goswami and Brown, 1990) or more candidate responses for the D item.

For simplicity, we simulate performance in binary choice verbal analogy problems, where only two candidate D responses are provided (denoted henceforth as A:B::C:[D1|D2], where D1 and D2 are the two alternatives). This is sufficient for the scope of behavioral phenomena we consider. We believe this focus on a single type of problem, together with the integration of both developmental and neuropsychological constraints, is a good first step for the development of an account in which verbal (and perhaps other forms of) analogical reasoning is viewed as an emergent consequence of reliance on learning and distributed representations. As such, our model complements other approaches which aim to address a broader range on analogical reasoning processes within the framework of mechanisms specifically constructed to support analogical reasoning (Hummel and Holyoak, 1997; Morrison et al., 2004; Doumas et al., 2008). We study the development of analogical reasoning as a consequence of knowledge acquisition and examine the special role of word associations. We suggest that word-association statistics complement the role of learning in explaining developmental patterns such as the relational shift seen in cognitive development from associative responses to appropriate relational responses. Also, we investigate the role of word-associations in performance following frontal or temporal damage, and explain how associative responding returns after frontal damage and the deterioration of cognitive control. Our theory is far from addressing all of the findings in the very broad analogical reasoning literature. However, we argue that we bring together findings from the more limited domain of forced-choice verbal analogy problems that have not been jointly considered before and provide an emergentist alternative to classical approaches to solving such analogy problems. Extensions to our framework will be required to address the full range of analogical reasoning paradigms.

In the rest of this introductory section we review the key findings that we consider to be important for the development and deterioration of performance in verbal analogies. Our model provides an integrated qualitative account of these findings. In section 2 we describe the architecture and representational assumptions of our model in detail. In addition, we explain the training process and testing of the model in analogy questions. In section 3 we demonstrate the results of our simulations. Finally, in section 4, we discuss the achievements and shortcomings of our model, compare it with other models in the literature and consider extensions to address a broader range of analogical reasoning situations.

### 1.1. Key findings

#### 1.1.1. The role of knowledge acquisition

Early developmental theories of analogy-making attributed developmental changes in performance to a domain-general progression through a series of stages. Piaget et al. (1977) found uncertain evidence of analogical reasoning in children from 5- to 12- years old. These findings for incompetence of analogical reasoning at these ages were aligned to Piaget's more general account of the development of reasoning. Similarly Sternberg and Nigro (1980) suggested that children's strategies shift from associative responding in early ages to relational reasoning through domain-general changes. However, Goswami (1991) has argued that these theories underestimate children's analogical reasoning abilities and the influence of the environment.

Precursors of analogical reasoning have been noticed in children in early ages from infancy in simple problem solving studies (Crisafi and Brown, 1986; Brown, 1989). Additionally, children in the ages 3–6 show competence in analogical completion in traditional forced choice analogy studies (Goswami and Brown, 1989, 1990; Rattermann and Gentner, 1998), contradicting Piaget's earlier findings. In the Goswami studies, the materials were chosen to be familiar to children. Thus, the conclusion was that what guides analogical development is experience with the items and relations involved, instead of a change in a domain-general mechanism. The Goswami and Brown (1989, 1990) finding that the ability of children to complete analogies within familiar domains, compared to the incapacity in the Piagetian studies (Piaget et al., 1977), suggests that the capacity for analogical reasoning is not based on a domain-general capacity for formal operations, but depends on the amount of experience that children have within specific domains of knowledge.

#### 1.1.2. Word associations and the relational shift

A number of factors may affect children's responses in forced-choice analogy problems. Sternberg and Nigro (1980) suggested that children's preferences in problems of this type are initially associative. Achenbach (1970, 1971) designed a task to test individual preferences on relational versus associative strategies. In an A:B::C:[D1|D2] task used to distinguish analogical from associative responding, the candidate D choices contain, among others, the correct analogical choice and at least one choice that is more or less associated to C than the correct response. For example in the PIG:BOAR::DOG:[WOLF|CAT] analogy problem, the correct response would be the WOLF. But the foil CAT, which has higher semantic association with the word DOG than WOLF has, can be used to test the ability to respond analogically despite the presence of semantic distractors. Sternberg and Nigro (1980) showed that the response speed and errors of 9- and 12-year-olds depended on the degree of association between candidate D terms and C terms in the analogies, thus they concluded that younger children rely on associations while older children rely on relational matching.

Goswami and Brown (1989) has argued, though, that these relations were hard for the children to handle and proposed that children rely on associations when there is not enough knowledge of the domain. Taken together, the findings suggest that, despite the primary effect that domain-specific knowledge has, the role of word association should not be disregarded. Gentile et al. (1969) discovered that word pair association factors can explain a large portion of the variance in analogical responding of university students, who could also be primed to respond associatively. Recent studies have also highlighted the influence of word associations in analogy completion. Thibaut et al. (2011) have shown that analogies constructed with pairs of weakly semantically associated items were harder for children with inhibition problems. Also, in a neuroimaging study of verbal analogies, Bunge et al. (2005) showed that strong associations in the A:B part of the analogy significantly improved performance in the analogy completion task. This suggests that the more familiar the A:B relation the easier the comparison with the C:D term is.

In all cases semantic association seems to play an important role which either facilitates or inhibits correct analogical response, according to the relative strength of the association between the C term and correct vs. the incorrect alternative.

#### 1.1.3. Neural basis of analogical reasoning

Given the centrality and complexity of analogical reasoning it is unsurprising that several brain areas, associated with various cognitive processes, are involved in analogy-making. Specifically, cognitive control and semantic retrieval processes are involved. Bunge et al. (2005) showed activation of distinct cortical areas in association with component processes of analogical reasoning (semantic retrieval and relational integration).

A large number of neuropsychological (Stuss and Benson, 1984; Shallice and Burgess, 1991; Duncan et al., 1995; Waltz et al., 1999) and neuroimaging studies (Baker et al., 1996; Prabhakaran et al., 1997; Osherson et al., 1998) have implicated prefrontal cortex (PFC) in complex and high-level cognition such as reasoning. Waltz et al. (1999) found that patients with frontal lobe damage had impaired performance in the more complex trials of the Ravens Progressive Matrices test (i.e., when more than one relation had to be integrated), a test that is cognitively similar to analogy tests. Mediation of the PFC has been found also in analogical reasoning tasks. Wharton et al. (2000) showed evidence for activation of the left dorsomedial prefrontal cortex (Brodmann's area 44 and 45) in geometric analogy problems.

Despite the evidence of activation of the PFC in analogical reasoning its exact role is still unknown. In non-analogy studies, Cohen and Servan-Schreiber (1992), by reviewing the deterioration of performance of schizophrenic patients in attentional (Abramczyk et al., 1987; Cornblatt et al., 1989) and linguistic (Chapman et al., 1964) tasks, suggested that the PFC plays an essential role in maintaining an internal context representation in a form that can constrain processing task-relevant input. Interestingly, Chapman et al. (1964) showed that schizophrenics could not interpret correctly a weak meaning of an ambiguous word even if the context of the sentence provided clear evidence for disambiguation. Instead, patients demonstrated meaning-frequency effects, preferring the more frequent meaning of a word over the contextually-appropriate meaning. Cohen and Servan-Schreiber (1992) provided simulations that captured this effect by lesioning a model component that corresponded to the prefrontal cortex. The PFC may play a similar role in allowing the correct alternative to be selected in A:B::C:[D1|D2] problems. Let us consider the concrete example we presented previously: In the PIG:BOAR::DOG:[WOLF|CAT] case the ability to pick the relationally appropriate response WOLF may depend on the PFC to maintain an internal representation of the A:B “context” to help override the strong association between DOG and CAT. The A:B part of the analogy is what provides the appropriate context for picking the analogically correct response.

In addition to the prefrontal cortex, temporal areas are argued to be important to verbal analogies, given their importance for semantic tasks (Hodges, 2000). The anterior temporal cortex (particularly in the left hemisphere) is argued to be important for verbally transmitted conceptual knowledge (Martin et al., 1996; Mummery et al., 1999).

The importance of these cognitive processes becomes apparent with the study of frontotemporal lobar degeneration (FTLD) patients. FTLD is a regional neurodegenerative etiology of dementia. A main classification of FTLD patients can be done, according to the primary locus of damage, which can be either in the frontal or in temporal areas and especially in the anterior temporal areas. Morrison et al. (2004) compared frontal and temporal FTLD patients' performance with that of control subjects. In a forced binary choice analogy task they found that both temporal and frontal damage patients made more errors than control participants. Since the choice was binary, one choice (called here D1) was correct and the other (called D2) was incorrect. The relative association of the C:D1 pair compared to that of the C:D2 was called the *Semantic Facilitation Index* (SFI). This Semantic Facilitation Index took positive, zero, and negative values. The sign of the index was based on an approximation of the difference between the C:D1 association and the C:D2. Frontal damage patients performed well with positive SFI problems (C:D1 association stronger than C:D2 association, where D1 is assumed to be the correct response), but their performance was impaired for equal SFI and especially negative SFI items, in which the incorrect choice had a higher association with the C term. Temporal patients, on the other hand showed overall depressed performance, and were less affected by SFI.

Table 1 summarizes the key findings that we consider to be important for treatment within an integrated mechanistic account. We believe that any framework of analogical reasoning needs to follow a knowledge-acquisition approach in order to address the overall role of experience in solving verbal analogy problems. Specifically, we highlight here the role of association strength (the environment's statistics) on performance. We suggest that people's ability to use the context provided by the A:B pair depends in part on prefrontal integrity to maintain a representation of this context and in part on prior experience, and that a by-product of this experience dependence is that the retrieval process is either facilitated or inhibited by the relative association of the C item with the correct alternative as opposed to the incorrect response. Our model qualitatively integrates and simulates these findings.

**Table 1:** **Key findings of verbal analogies**.

**DEVELOPMENT OF VERBAL ANALOGICAL REASONING**	
1.	Experience in relational knowledge is a key force behind the development of analogical reasoning (Goswami, 1991)
2a.	There is a shift in analogical responding from associative strategies to relational reliance (Sternberg and Nigro, 1980)
2b.	This relational shift must be supported by knowledge acquisition (Goswami and Brown, 1989)
**THE NEURAL BASIS OF VERBAL ANALOGIES**	
3a.	The prefrontal cortex has been implicated in analogy and analogy-like imaging studies (Waltz et al., 1999; Wharton et al., 2000)
3b.	A putative role of PFC is to maintain an internal representation of task context (Cohen and Servan-Schreiber, 1992)
3c.	Frontal lobe lesions cause strong influence of associations in responding (Morrison et al., 2004)
4.	Temporal lobe lesions cause general impairments in performance regardless of task relative associations (Morrison et al., 2004)

### 1.2. Modeling framework and design goals

Our model belongs to the Parallel Distributed Processing (PDP) tradition (McClelland et al., 1986; Rumelhart et al., 1986a). Connectionist networks embody characteristics that are appealing for relational representation such as gradience in representation, interactivity in a bidirectional manner between units allowing mutual satisfaction of constraints, nonlinearity, and adaptivity (McClelland, 1993). The principle of graded representations is essential for being able to represent a graded performance of analogical reasoning instead of an all-or-none approach where a comparison is or is not analogically appropriate. In addition, allows developmental explanations based on knowledge acquisition. Connectionist networks are accompanied by learning algorithms that allow them to modify their weights, and hence their knowledge, with experience.

In line with the gradient character of our framework, one goal of our model was to address gradience in analogical reasoning. We argue that analogies can be drawn between pairs of items that have similar, instead of completely identical, relations. For example we suggest that the relation between DOG and PUPPY is more similar to the relation between CAT and KITTEN than it is to the relation between RIFLE and PISTOL, but also that both the DOG:PUPPY::CAT:KITTEN and DOG:PUPPY::RIFLE:PISTOL analogies can be valid, even though the relations vary in their degree of similarity. By using distributed relational representations we are able to solve relational problems even for cases where the relational representations are similar but not identical.

Second, our model is motivated by a desire to allow the relation retrieved between A and B to be affected by the rest of the analogy problem. As argued by French (2008) one has to consider both parts of the analogy to figure out which relation is the most appropriate. Considering our previous example, one cannot be a priori certain that the relevant relation between DOG and PUPPY is that of kinship, size-relation or anything else before the candidate relations are constrained by the C:D terms. Thus, we consider interactivity during relation-retrieval to be crucial for our theory.

Before turning to the details of our model, we note that our work builds on two previous modeling efforts. Leech et al. (2008) proposed a learning based model that served as one of the main inpirations for our approach, demonstrating how learning could explain aspects of development of analogical reasoning abilities. Morrison et al. (2004) offered a model of the pattern of neuropsychological deficits seen in FTLD within the LISA model of analogical reasoning (Hummel and Holyoak, 1997). Our model differs from both of these earlier models in several ways, and is the first to address both the developmental data and the neuropsychological findings within the same model. In the discussion we consider similarities and differences between the models in more detail.

## 2. Materials and methods

### 2.1. Neural network model

#### 2.1.1. Architecture and representation

Under our approach verbal analogies are a by-product of simple relational learning. A cognitive agent is exposed to item1-relation-item2 triples. It learns to associate the items with each other, such that the presentation of the two items tends to result in filling in the relation. This relation, in turn, may then work together with the presentation of one of the two items to constrain the retrieval of the other item. Our model draws on related early work by Hinton (1981). Hinton's effort embodied the same computational principles and the same psychological content. In an effort to implement semantic networks in parallel hardware, Hinton introduced a network very similar to the one proposed here, though at the time the learning machinery available for training such networks was more primitive.

**Figure 1:**
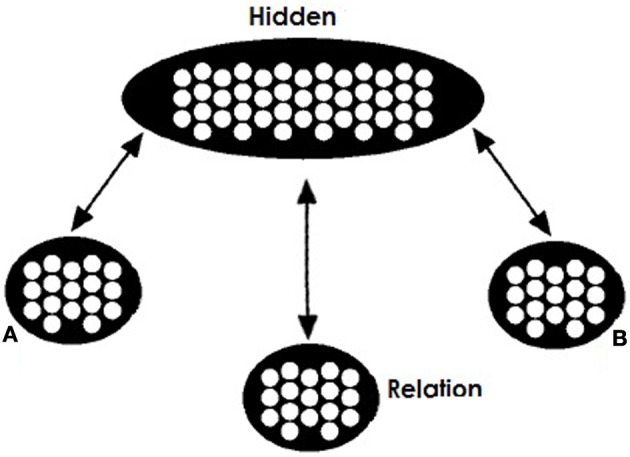
**Model's training architecture**. The network consists of two visible pools (A and B) for the two concepts in a relation, a visible pool (Relation or R) for the relation between them, and a hidden pool. Connectivity between pools is bidirectional.

Our network's training architecture is shown in Figure 1. Similarly to Hinton's network there are two visible pools for the role-fillers of a relational triple (A and B), a visible pool for the relation (R) and a hidden integrating pool. All three visible pools are connected with bidirectional projections with the hidden pool.

Objects in the A and B pools are represented in a localist manner. In contrast, representation in the Relation pool is distributed. We acknowledge that localist coding does not allow the network to capture the subtleties and effects of surface similarity between concepts, but reduces the complexity of learning for the network. For the Relation pool patterns of activation correspond to specific relations with similar patterns representing similar relations. Representations for a relation correspond to activations of 0 and 1. Each unit is assumed to correspond to semantic or visual features of the relation. In our simulations relations that are seemingly the same or can instantiate a valid analogy are assumed to come from a shared prototype pattern. For example the relation for the pair PIG:BOAR will be very similar to the relation for the pair DOG:WOLF since they both are distorted instances of a prototype pattern approximately corresponding to the relation “domesticated form of.” Importantly, these two instances will be similar but not identical. Activation of appropriate representations in these three pools corresponds to a specific relational fact. We will call these facts *propositions* and henceforth denote them as A:R:B or A:B with the relations being implied. Of course we hold that both items and relations involve distributed representation—we use distributed relation representations to underscore that the relation (like items) are likely to vary across cases that might sometimes be labeled as the same, and to demonstrate that relations need not be identical for analogical reasoning to succeed.

#### 2.1.2. Training

We train the network to complete relational propositions when given any 2 of a triple's elements as inputs. We use the backpropagation-through-time (Rumelhart et al., 1986b) learning algorithm as implemented in the pdptool simulation environment [version 2.07, McClelland (2012)]. As it learns the associations between objects and relations, the model assigns to each input a stable pattern of activity across the hidden units. For each training epoch a set of propositions is presented to the network. This set of propositions corresponds to the network's environment. Each proposition (i.e., each A:R:B triple) appeared many times within each epoch. One third of the time, the A and B items were presented as input; in another third, the A and R items were presented as input; and in the final third, the B and R items were presented as input. In all three types of cases, the network had the task of filling in or completing the third member of the triple. Also each input combination (i.e., A:B, or A:R, or B:R) for a proposition can appear multiple times within an epoch. This number of times is called the proposition's *frequency*. We assume that the frequency of co-occurrence of items within propositions is an important contributor to the strength of their association, an idea well grounded in psycholinguistics (Spence and Owens, 1990). Of course we don't argue that associative value is captured only by co-occurrence frequency, but instead that it is being sufficiently approximated and on the same time allows us to address our questions on a very simple neural network. We leave the details of the model's environment for the Simulations section and a complete description is given in the Appendix.

#### 2.1.3. Testing

Our model is not trained on analogies *per se*, and we show that the ability to complete analogy problems can emerge from our training architecture. One way in which this might work would be to imagine that the network is first presented with A:_:B, and fills in the appropriate relation R; and that R or a trace of it persists after removing B and replacing A with C, allowing completion of the C:R:_ triple. Such an approach would be similar to the “relational priming” framework (Leech et al., 2008), which is grounded empirically on findings suggesting that analogies occur spontaneously (Goswami and Brown, 1989; Pauen and Wilkening, 1997; Tunteler and Resing, 2002). However, instead of using priming as a mechanism for retrieving the A:B relation first, and later use that to infer the D term, we suggest that the brain may possess the ability to simultaneously represent the A,B,C, and D terms of the analogy, and can use them all together to find a common relation that completes both the A:_:B triple and the C:_:D triple. Note that we do not claim that the brain's architecture has this capability solely to solve verbal analogies problems, but that, in general it possesses the capability of allowing mutual constraints to influence completion of neighboring propositions, just as mutual constraints can shape the perception of letters in visual letter perception (Rumelhart and McClelland, 1982). For present purposes we rely on this capability only for analogical reasoning, however.

**Figure 2:**
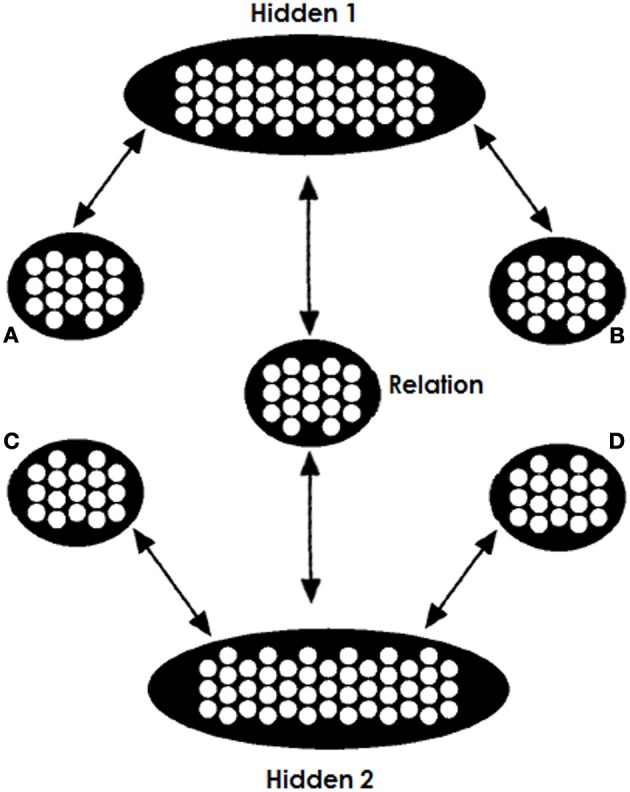
**Model's testing architecture**. Two copies of the trained network share a common relation pool, so that both the A and B terms and the C and one candidate D term jointly constrain the search for a relation. While the A, B, and C items are clamped for the entire testing process the D items are clamped only at the beginning. We run two tests, one for each candidate D item; the D alternative with the strongest “echo” of activation at the end of testing is chosen.

The architecture we use is shown in Figure 2. The network consists of two copies of the trained network, sharing a common relation pool. The weights between A and Hidden1 (H1) are identical to the weights between C and H2; the weights between B and H1 are identical to the weights between D and H2; and the weights between H1 and R are the same as the weights between H2 and R. This way, the network takes advantage of its experience. Finally, one more refinement is required. In the thought experiment where one clamps to both the top and bottom part the same A:B inputs (denoted as A:B::A:B), the net input arriving at the Relation pool is double the input that it was trained to receive. What that means is that the net input that this pool receives departs from its experience and has essentially double the magnitude. For this reason we have halved the contribution of the H1-to-R and H2-to-R projections.

It is important to stress that we do not assume the brain literally contains two copies of the identical network, sharing the relation pool between them. We do, however, assume that both parts of an analogy problem can access connection-based knowledge at the same time and can mutually constrain each other, something that is made possible by this architecture. We assume that this ability is part of the general cognitive machinery that allows the interpretation of each of two items to be constrained by the other, even if one is presented first. A model with some relevant properties was previously proposed by McClelland (1986).

When the network is clamped with A,B,C, and D representations, the two parts of the network will try to fill in the R pool the relations associated and learned for both of these two pairs of objects. Activation in the relation pool will depend upon the activation of the Hidden1 and Hidden2 pools. The Hidden1 pool will acquire a representation learned for the A:B input and Hidden2 will acquire a representation learned for the C:D input. Thus, Hidden1 will push the Relation pool toward representing the relation between items A and B and Hidden2 will push toward the relation between C and D. The more similar the two relations are, the greater the goodness of the network's state. Since all inputs are hard-clamped, the consistency of the Relation pool does not affect activation in the A,B,C nor D pools. However, when the D item is unclamped the consistency of the two relations and the goodness of the network's state will affect activation in pool D. If the two relation are similar, then the completed Relation with the C term will support activation of that D term. However, if the two relations were less similar, then the filled relation will not resemble the relation between C and D and thus the D item will not be supported by activation in the Hidden2 pool (we use below an example to make this idea clearer).

The test procedure we used is similar to one used by Dilkina et al. (2010) in a lexical decision task.^1^. For each analogy question we conduct two test trials. We clamp the A, B, and C items in their corresponding pools for the entire test. Each of the D alternatives is clamped on the D pool for some processing cycles, then removed for several more cycles, and the residual activation of the D unit that was initially activated is then recorded as a measure of the strength of the “echo” produced by that alternative. The alternative with the strongest echo is chosen as the network's response.

In summary, for a given analogy question A:B::C:[D1|D2], the process below is followed: The model calculates separately how good the A:B::C:D1 and A:B::C:D2 analogies are and compares their goodness to find the network's response. For each analogy we clamp the A, B, C, and D terms to their corresponding pools for a few processing cycles. During this phase, activation is spread over the network. Of interest is the fact that the A and B terms in the top part of the network push activation in the Hidden1 and the R pool as the network has learned to do in training. The same happens for the C and D terms. If the two pairs (A:B and C:D) share similar relations then the top and bottom part of the network will pattern-complete in the R pool similar representations. If they have different relations then the two parts of the network will push dissimilar representations in a resulting “meaningless” representation. After the first phase, we unclamp the D term (but keep all others clamped) and let the network process a few more cycles. By unclamping here we mean that we stop hardcoding input activation but keep the pool's state as it was without flushing it to zero. At this second phase, activation is still spread. Of interest is the activation in the D pool. The bottom part of the network has the C term clamped and now has a relation partially filled. Whether this filled relation was consistent (A:B and C:D similar) or inconsistent (A:B and C:D dissimilar) will determine how the bottom part of the network will allow the D activation to be maintained (echo measure). For consistent relations between the two parts the D term maintains higher activation. The model then chooses the D term with the higher maintained activation.

**Figure 3:**
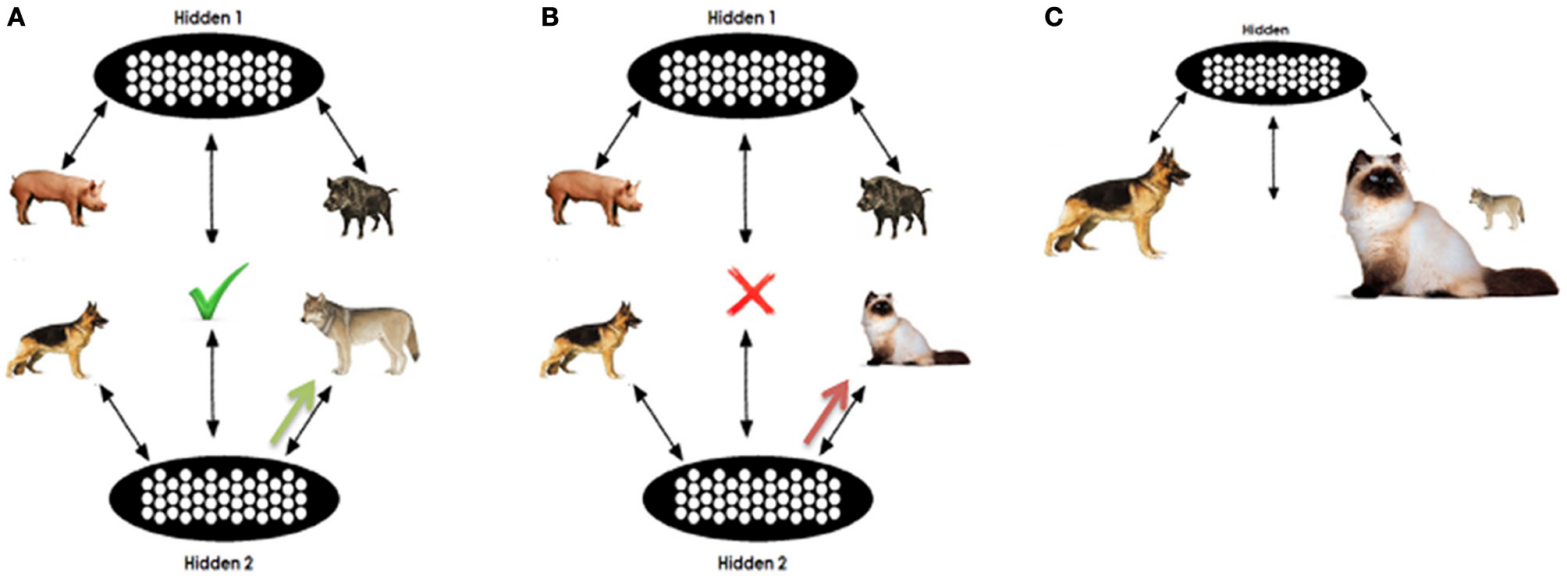
**Illustrative analogy question. (A,B)** Sketch analogy testing in the question PIG:BOAR::DOG:[WOLF|CAT]. The concept WOLF will initially support consistent pattern completion in the R pool. When unclampled, the partially completed pattern will support residual activation in the D term opposed to the CAT. However, as shown in **(C)** the higher association of CAT with DOG provides an associative advantage when incomplete or insufficient information is clamped in the relation pool.

An example is given in Figure 3. As mentioned previously, for each analogy question we perform two separate tests on the network (one for D1 and one for D2). In our example, in one test we clamp PIG, BOAR, DOG, and WOLF in pools A,B,C, and D respectively; in the other test, CAT is clamped on the D pool instead of WOLF. The D item will be clamped for a few processing cycles. According to the degree of training that the network has received the activation in the top part will push the R pool's representations toward activating the relational pattern of the proposition PIG:BOAR. On the other hand activation on the bottom part will push the R pool toward the C:D relation. An approximation of the joint representations for the A:B and C:D pair is filled in the relation pool. In the case of DOG:WOLF the relation is very similar to the PIG:BOAR, opposed to the case of DOG:CAT. Thus, the R pattern completed in the DOG:WOLF case is more consistent with the DOG:WOLF proposition as it appears in the training set, opposed to the DOG:CAT proposition. This way, the WOLF unit is expected to maintain higher echo (activation at the end of processing).

However, it is important to note that such a behavior depends on the stored weights that the network has acquired and the extent to which acquired knowledge can support relational retrieval versus free association. D activation will also depend on the frequency and association of each of the D terms with the C term, given the presence of C. In our example, CAT is trained more frequently with the word DOG than is WOLF, thus the response CAT gains an advantage this way. Representational similarity facilitates analogical responding, but frequency facilitates associative responding, regardless of which alternative is the relationally correct response. Our expectation is that the interactions between these two forces will support the correct response in tasks where the correct is strongly associated with the C term and will prevent correct responding in tasks where the correct response is weakly associated, but that as training progresses, the network's encoding of both low and high frequency associations will become sufficiently robust that the relational similarity will allow correct responses, regardless of relative frequency.

#### 2.1.4. Effects of frontal and temporal damage in frontotemporal lobar degeneration (FTLD)

***2.1.4.1. Frontal damage.*** Following the ideas of Cohen and Servan-Schreiber, (1992) we assume that frontal damage diminishes an individual's ability to maintain context information—here, the representation of the A:B item—needed to constrain the C:[D1|D2] decision. One way PFC might do this is to regulate the overall activation of the hidden units mediating the A:B association. Accordingly, we treated frontal damage as reducing an overall biasing input to the hidden units in the A:B part of the network. This leads to a reduction of activation in H1 pool, impairing the ability of the A:B pair to influence the pattern of activation on the relation units, thereby causing the network to operate approximately as in Figure 3C. There are other possible ways in which PFC damage might reduce the contribution of the A:B association to constraining the specification of the relation between C and D which would likely have similar effects, and it is possible that different frontal syndromes (e.g., FTLD, schizophrenia) might produce such an effect in slightly different ways.

***2.1.4.2 Temporal damage.*** Anterior temporal damage in the network is much more straightforward. The role of the anterior temporal lobe is to allow the completion of propositions—in our case, the filling-in of the missing relations between the presented items. This mechanism is mapped to the pattern completion processes of the two parts of the network. Since all projections in the network are essential for pattern completion we will assume that random loss of connections corresponds to anterior temporal damage. The approach of randomly removing connections (set their weights to 0) has been followed by Rogers et al. (2004) for lesioning a model of semantic memory. Relying on our assumption that both parts of the analogy draw on the same underlying connection-based knowledge, we removed connections from one part of the network (A:B part) at random according to a specified probability, then copied the projections from the lesioned part of the network to the complementary (C:D) part, so that the lesion was identical for both parts of the network.

### 2.2. Simulations

We ran two simulations. The first was intended to demonstrate how the relational shift emerges within a single-purpose learning network. For our second simulation we used the trained networks of the first simulation and applied lesion to demonstrate how our model accounts for frontotemporal lobar degeneration. The two simulations use the same training set, which we describe in the following section. For each simulation we trained five networks with randomly initialized connection weights and their own randomly generated training environments.

#### 2.2.1. Relational patterns

**Figure A1:**
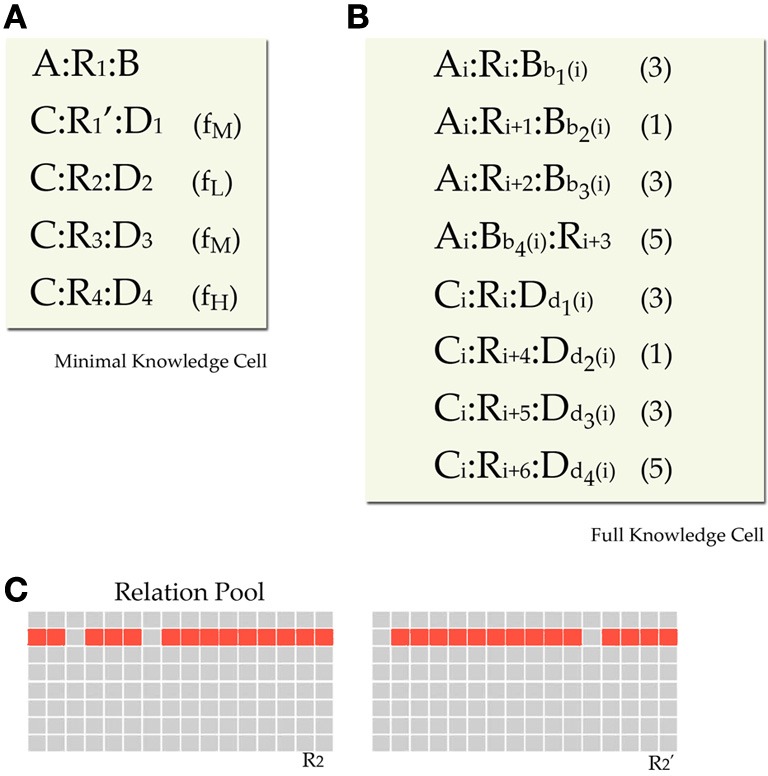
**Model details. (A)** Minimal cell design as described in Materials and Methods. **(B)** Final design of a knowledge cell augmented with propositions that serve counterbalancing purpose. Each cell has 8 propositions (one source, one correct target, three foils, and three additional). All A, B, C, and D items are reused in various propositions in various cells for counterbalancing frequencies. All A items have the same frequency between them by being used in all first 4 propositions of a cell. Similarly for C items in bottom 4 propositions. All relational prototypes appear in all 8 rows as 8 different instances. A prototype appears in two rows (source and target) in its respective cell and shift in other cells. Items B and D appear in their respective 4 rows in different cells. We constrain them so as to appear in different rows in different cells and not reappear associated with the same A/C or R items. **(C)** Activation of two instances (*R_2_1__* and *R_2_2__*)derived from the same prototype **R2**. Each 16 units are dedicated to a relational prototype. For each instance we turn off 2 randomly chosen unique units.

The relational pattern representations were generated as follows. Relations in the training set come from 8 different relational prototypes, consisting of 16 active units out of the full set of 128 relation units. **R_i_** refers to one of the relational prototypes. Two different prototypes (thought of as corresponding to very dissimilar relations) have no overlap at all on their set of active units. However, relations generated from the same prototype have 12 units in common. Specifically, an instance of a relation is obtained by turning off two of the units of the active units of the prototype. The turned off units are necessarily different across instances (See Figure A1C for two instances of the **R_2_** prototype).

#### 2.2.2. Training environment and parameters

**Figure 4:**
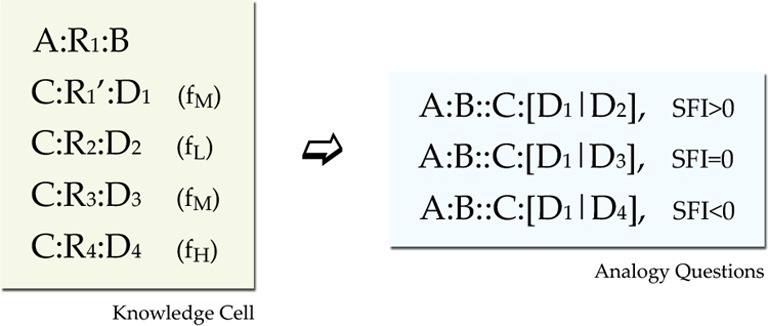
**Training environment design**. Minimal design for a knowledge cell. The source proposition provides the A:B part of the analogy question, the C:D1 provides the correct response and there are three foils with various associations (shown in parentheses as Low, Medium, and High training frequency). Further details of the environment design are presented in the Appendix.

The training environment for each network consisted of blocks of propositions called *cells*. Each cell was designed to provide three analogy questions. One with positive SFI, one with neutral, and one with negative (recall that SFI is defined as the relative association of the correct response with the C term compared to the association of the incorrect response with the C term). For satisfying such a constraint, each cell should minimally have the format seen in Figure 4. In this format we have a basic *source proposition* A:R1:B, one *relational target* proposition C:R1′:D1, and three *foil target* propositions C:R2:D2, C:R3:D3, and C:R4:D4, one *weak*, one *moderate*, and one *strong* (Note that each relation in the above propositions is an exemplar from a different prototype, except that R1 and R1' are exemplars form the same prototype). This set gives three analogy tests (SFI>0: A:B::C:[D1|D2], SFI=0: A:B::C:[D1|D3], and SFI<0: A:B::C:[D1|D4]). The three kinds of test within a cell are a result of the frequency variation that reflects association variation of a word (C) with several other words (D1, D2, D3, D4). Such a minimal design confounds frequency of association with frequency imbalances in the rates of occurrences of relations and items. Seemingly, a word D4 that has a high-frequency of co-occurrence with the word C for example, seems to have overall higher frequency of occurrence. To fix that, by keeping the structure behind this basic design that yields 3 analogy questions, we counterbalanced global frequency of training by reusing items and relations in propositions of various frequencies across cells (See Appendix for details on the imbalances and our scheme for counterbalancing).

## 3. Results

### 3.1. Simulation 1: relational shift

We trained 5 randomly initialized networks with 5 randomly generated training sets (as described in the Appendix) for 350 epochs. The average error measure was almost zero at the end of training. However, what is important is not performance on the relational propositions, but on the analogy questions (higher echo of D1 vs. foils for each cell). At 350 epochs the networks had an average correct performance in the analogy tests of 0.97.

**Figure 5:**
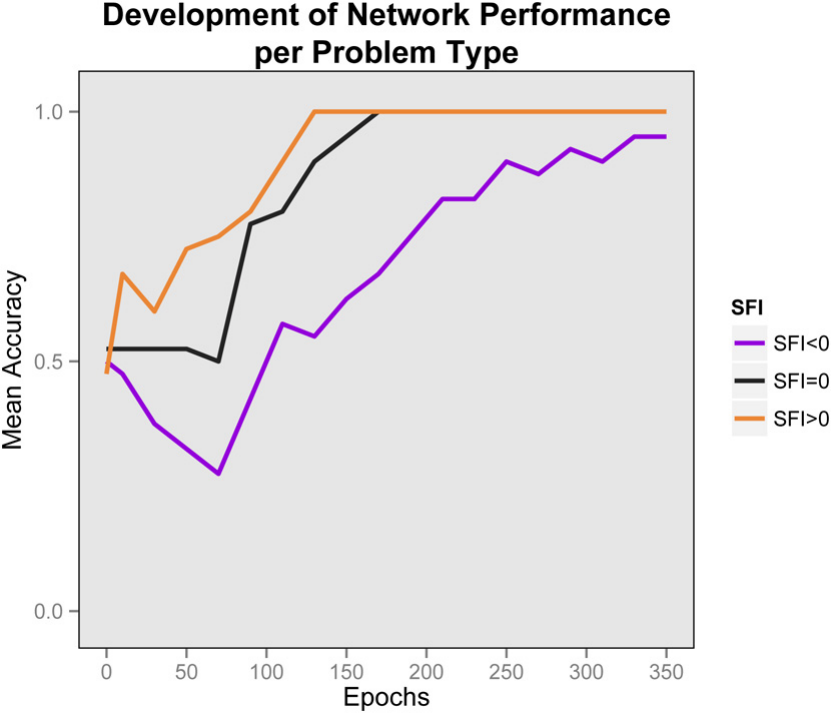
**Development of performance by problem type**. Average performance of networks, by problem type in time. The relational shift is apparent by the early bifurcation of the plotted lines according to problem type combined with the convergence to correct responses later. Chance is at 0.5. Early in training, performance is below chance for the negative SFI problems and higher SFI problems are learned faster. Later, performance is improved for all problem types.

One important focus of interest is the development of this performance through time. We sampled performance every 10 epochs (initially all networks had performance at chance). In Figure 5 we show the development of performance by problem-type. It is obvious that for all problem-types performance improves with knowledge-acquisition. Importantly, the networks learn to solve problems with higher SFI before other problem types, and is impaired early on for problems with lower SFI. The fact that performance is significantly below chance in the negative SFI condition indicates that responding is primarily determined by differences in association strength early on. Higher association of the correct response facilitates performance, while for the negative case, lower association inhibits performance and the networks fall below chance even after 10 epochs of training. Performance in the negative SFI condition starts to show improvement at approximately 80 epochs of training and the networks became relational even for the negative SFI case after 130 epochs of training, when the acquired knowledge allows the testing process to overcome the prepotency of the high-association foils. This pattern can be described as a relational shift, since the early bifurcation can be attributed to a reliance on word-association, while later performance relies on relational knowledge. Note that this occurred, even though the network was never trained to carry our analogical reasoning. Once both parts of the analogy are highly familiar, their mutual constraint outweighs associative responding.

### 3.2. Simulation 2: FTLD

**Figure 6:**
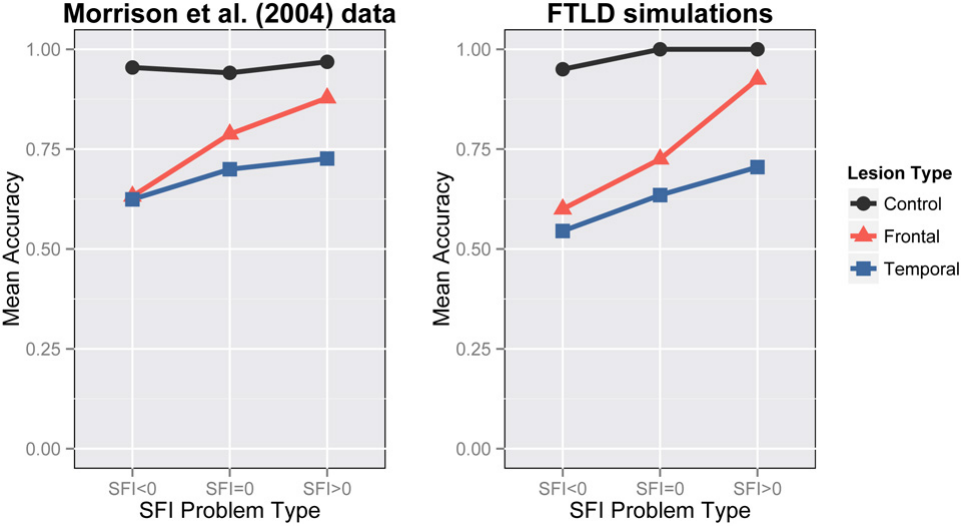
**Frontotemporal lobar degeneration simulations**. Performance on analogy problems per problem-type for various lesions. **Left**: FTLD data from Morrison et al., 2004 (Reprinted with author's permission). **Right**: Simulation 2 results.

Our simulations aim to capture the same key characteristics that Morrison et al. (2004) classified as important (Figure 6-Left):

1. control participants showed good performance at all levels of SFI,
2. frontal lobe patients showed depressed performance for lower SFI problems,
3. temporal patients showed depressed performance, and
4. both frontal and temporal patients exhibit a SFI effect, which is bigger for the frontal patients.

We lesioned the 5 networks of Simulation 1 at 350 epochs of training. We applied either a frontal lesion as a reduced bias in H1 pool or a temporal lesion as random loss of connections. The bias in the H1 units was reduced from −2 to −6.5 for the frontal lesion and connections were removed with .42 probability for the temporal lesion. Since the temporal lesion was randomly applied, we generated 5 lesioned versions of each network resulting in 25 networks with temporal lesions in total. Our network accounts for the interaction of lesion-type with problem-type. It is obvious that the negative SFI-problems are impaired compared to neutral and zero after frontal damage and there is an overall degradation of performance after temporal damage (Figure 6). When frontal damage is applied to the model, evidently the model loses part of its ability to respond relationally and moves to associative strategies. This happened because the H1 pool loses its ability to maintain a representation of the A:B proposition. Hence the relation pool is influenced predominantly by the C:D part of the analogy. In the bottom part of the network there was only a constant input coming from the C pool. Thus, given the lack of other constraints, the most natural reaction is to complete the patterns that are more frequently trained with C. This is how associative responding emerges in our network. We consider this a very important implication of our model that is consistent with previous work on the prefrontal cortex (Cohen and Servan-Schreiber, 1992). In the semantic case, the network lost its ability to successfully complete the patterns. Hence, the whole process can be considered a noisy version of the control test.

As mentioned previously, there is a SFI effect in the experimental data for both frontal and temporal groups—the effect is larger for frontal than for temporal lesions, and this is captured in the simulation results as well. In both frontal and temporal cases the SFI effect is larger in the simulations than in the experimental data. We believe that the size of the SFI effect empirically will likely depend on a range of factors, including the degree of asymmetry of the word associations—our model appears to show a larger asymmetry effect overall than the experimental data. A possible reason for that could be the fact that the actual associations used in the experiment were less asymmetric than our model assumed.

## 4. Discussion

We aimed to provide a model of key findings of verbal analogical reasoning. Despite our apparent focus on a specific class of analogy problems we unified disparate findings related to normal performance, development, and deterioration of verbal analogical problem solving within a learning system that learns relationally-mediated associations. Our simulations were qualitative and aimed to explain key phenomena at an abstract level, however, the basic pattern of the findings were robust and consistent with the basic patterns seen in developmental and neuropsychological data.

We showed how a neural network trained solely on relational propositions can solve analogy questions by allowing both halves of the analogy problem to mutually constrain the selection of a relation. In addition we showed in accordance with Goswami's theories how knowledge-acquisition can drive the improvement in performance during development—we do not require the invocation of a qualitative change in processing but only the gradual buildup of relation-mediated associations as the basis for the so-called relational shift. Importantly, we showed how knowledge acquisition can interact with the environment's statistics in a complementary manner to explain the behavioral patterns observed during development. Specifically, we explained the shift of children's reasoning from associative to relational as a by-product of learning and pattern completion on a given architecture. The architecture assumes cognitive control components that attempt to use acquired contextual information for overriding prepotency of incorrect responses. We showed that early in training top-down contextual information was not enough for overcoming the prepotency of strong foil responses. However, after training, without any changes on the system's parameters or architecture, this phenomenon is diminished and the network learns to yield relationally appropriate responses. The same network, trained on the same training environment, explained the overall degradation of performance after temporal damage as the loss of connections responsible for pattern completion processes and explained the return of associative responding after frontal damage as the loss of capacity to maintain context-related information that guides the retrieval of the appropriate target representations.

### 4.1. Model limitations

Of course the interaction between frontal lobe development and knowledge acquisition is of great interest. It is important to note that our theory does not exclude frontal lobe development as a causal factor behind the behavioral pattern of analogical reasoning during development. On the contrary, executive-functions skills, attentional switching, and inhibitory control play very important and specialized roles in the development of analogy-making (Richland et al., 2006; Morrison et al., 2011; Richland and Burchinal, 2013). However, we argue that the developing frontal lobe synergistically with background knowledge cause the observed relational shift. Even if frontal-lobe development has its own trajectory we argue that it needs to exploit not only changes in frontal control functions but also acquired knowledge.

Our goal was not to provide a mapping from model components to brain areas. We do not believe that the two separate network parts reside in different brain areas, but instead that our architecture provides a neurocognitive explanation of the role of top-down contextual biasing. The frontal lobe, however, has a dual role in such a task. The one is to actively maintain goal-relevant contextual information (Cohen and Servan-Schreiber, 1992) for top-down biasing. The other is to guide attentional switching between what we model as two different networks (Hummel and Holyoak, 1997; Doumas et al., 2008; Morrison et al., 2011). While these functions are potentially somewhat different in nature, the extent of their separability is unclear and they may potentially share the same underlying neural basis. Damage in the frontal lobe in FTLD patients probably causes severe impairments in both functions. Our theory, however, deals with impairments only in the former. We acknowledge that impairments in attentional switching functions as well (a component function not explicitly included in our model) could play a role in the associative effects found in frontal patients. Moving toward neurally grounded models will help us understand how the plausibility-driven constraint of interactivity is actually implemented in the brain and how it is deteriorated with frontal damage. As a first step, we argue that our model is not incompatible with the switching function. In terms of the architecture shown in Figure 2 the top-down function would focus on actively maintaining the retrieved relation for the A:B pair of the analogy providing bias in the Hidden1 layer, as explained through the paper. We tried other forms of lesioning the top-down biasing function (e.g., impaired clamping in the A and B layers) and all had similar results, showcasing the importance of active maintenance of information in the A:B part of the network. Then switching control would be used to map this relation to the one from the C:D pair. This mapping could be done interactively by means of several continuous rapid switches of attention from one pair to another.

Our model did not aim to provide a fitted quantitative match for the data in the literature. While this is a goal for future work, we suggest that our approach is a useful, and perhaps necessary, first step. We were very concerned with potential confounds caused by stimulus-frequency constraints, so we prioritized counterbalancing. This allows us to be sure that our results depend on the co-occurrence frequency factors and not on the frequency of the items and relations that enter into these associations. Our design allowed us to counterbalance overall frequency of training of each word or relation. Of course, we don't argue that such counterbalancing is plausible and we believe that frequency of a specific item indeed plays a crucial role in analogy making (both in reality and in our theory). However, such questions were considered to be out of the scope of our current model. In future, it will be important to consider how such a framework can be extended to process more plausible data-sets.

A final limitation of our model is that it lacks an explanation of how the relational representations are learned and developed. We believe that relation representations change as a function of experience, but out current model lacks this property. Even if the environment provides invariants for many visual relationships (Doumas et al., 2008), we think it may be inappropriate to assume that a cognitive agent has available learned representations of complex relations like “is among the strongest” or “is the favorite student of”. Instead, a complete model of analogical reasoning should consider how these representations are learned and shaped by their exemplars. Our model provides a suggestive initial framework for capturing the interacting complementary role between a learning agent and it's environment, and provides a base on which further work can proceed to address this issue.

### 4.2. Comparison with other models

As mentioned earlier, our model is related to and inspired by the neural network of Leech et al. (2008). We believe our approach advances these author's relational priming approach in several different ways, some of which were circumstantial to the relational priming model and some of which were intrinsic to it. The nature of the theory for “relations as transformations” addresses intuitively only a small class of relational propositions, namely propositions that express causal transformations. Such causal relationships were used by Goswami and Brown (1989) in pictorial analogy tasks. However, our use of distributed representations allows for a more flexible representation of relations, giving us the flexibility to address a wider range of relation types and corresponding findings. As discussed in the peer-commentary of the relational priming Leech et al. (2008) paper, transformation upon relations that operate in a linear manner on item representations (as implemented on the relational priming approach) suffers from non-transitivity. Our flexible representation of relations does not come cost-free, however, since our theory lacks a complete description of how such representations develop.

Also, importantly, as argued by French (2008) a priming-based approach does not cover the need to consider both parts of the analogy before settling to the correct response. Our network interactively considers both parts of the analogy for completing the shared relation. Although it is likely that this interactivity was not necessary to account for the data we simulated, we nevertheless agree with French (2008) that such interactivity has a role to play in analogical reasoning. Our simulations also used similarity, rather than strict relational identity, as a basis for analogical reasoning. While we did not explore effects of variation in relational similarity, pilot results from preliminary simulations revealed that, indeed, a higher number of shared relational “microfeatures” (Hinton, 1981) is associated with higher levels of activation. We set as a future goal the implementation of more complete simulations that will more fully exploit the interactive and similarity-based features of our architecture.

On the other hand our model differs from the LISA approach significantly. Morrison et al. (2011) have recently considered the relational shift within the LISA theory. Admittedly, the LISA theory provides a much more complete framework for a vast array of findings related directly or indirectly to analogical reasoning. We believe our approach has an important benefit. Our cognitive control explanation (both for the relational shift and for the frontal lesion) is fundamentally different than that proposed in the LISA model. In our case, cognitive control is expressed as a top-down influence in the network's operation that does not directly inhibit irrelevant information. Instead, inhibition of alternatives naturally arises as a consequence of competition among alternatives; PFC serves primarily to maintain a representation of context, so that the mutual constraint between the A:B and C:D pairs can proceed, and the relational shift emerges within the network without any hard-coded domain-general changes but as a simple consequence of learning. In contrast to this, Morrison et al. (2011) argue that the relational shift is a result of the domain-general maturation of the inhibitory system that parametrically is hard-coded to change values during development. While maturation of the PFC is likely to play a role in maintaining context representations, we emphasize that experience is also likely to contribute to the relational shift during development. Our approach is emergentist and such a suggestion is very important in cognitive science (McClelland, 2010) as it eliminates the need for assuming specialized systems or hard-coded components. In addition, our framework provides unified simulations of both the developmental and neuropsychological findings, operating upon the same training set, something that is important for the plausibility of the theory. We believe and hope that the two classes of models will both contribute to the further development of mechanistic accounts of analogical reasoning.

### 4.3. Future work

We consider the potential of addressing findings in the cognitively related field of metaphor comprehension. Metaphor comprehension can be seen as the process of filling out the A and D terms of an analogy in which the B and C are given Turney and Littman (2003). Consider the metaphor “demolish an argument.” Comprehension of such a sentence can be seen as the process of inferring an analogy between two domains which the metaphor links. One can think of the analogy CRITICIZE:ARGUMENT::DEMOLISH:BUILDING. The statistical pattern completion properties of neural networks are appealing for such a task. Clamping the known B and C terms might lead to completing the unknown terms, given the constraints that the R pool will pose. Such a prospect further justifies our modeling choice for an interactive architecture.

Finally, we note that an extended version of the model might some day be applicable to non-verbal analogies problems of the type found on the Raven's progressive matrix test. Experience with propositional relationships expressed in verbal form is likely to be of relatively little importance for such problems. However, there is still a potentially important role for an interactive architecture such as ours, in which selection among alternative visuospatial relationships rather than verbal relationships is mutually constrained by the given items in the specified cells of the matrix and the alternative choices provided for the completion of the missing cell. In such a model we would expect that we would observe, and be able to simulate, a role for factors similar to those at work in the current model, including relative familiarity of the correct relation and extent of cognitive control needed to allow a relation common to different rows or columns of the matrix to win out in competition with others.

### Conflict of interest statement

The authors declare that the research was conducted in the absence of any commercial or financial relationships that could be construed as a potential conflict of interest.

## Footnotes

^1^We explored a variety of different alternative decision criteria. The first was the echo of activation (residual activation after unclamping) as mentioned before. Alternatively, we left the D terms clamped for the entire processing and used the net-input at the end of processing as a decision criterion. Finally, we did not clamp any of the D items and selected the item with the highest activation at the end of processing. The network's responses were the same for all three different criteria, thus we do not report any simulations for the other two decision-criteria. We preferred the echo criterion because it expresses the close-ended nature of the task, without allowing the network to consider irrelevant alternative options. It also lies somewhere between the two alternative considered options allowing for both open-ended retrieval processes (echo after unclamping) and close-ended binary choice (necessary decision of higher echo among two alternatives).

## Appendix

### Training set design

As mentioned in the Materials and Methods section we used *cells* of knowledge. Each network's environment consists of 8 cells of knowledge. The 8 cells have the same basic structure (as shown in Figure A1A and in the main text), but they are augmented with additional propositions. The complete design is explained here. The network's world consists of 32 items and 8 relational *prototypes* (i.e., types of relations). Among the 32 items, 16 appear in the first slot of a relational proposition (8 of them act as A items in propositions and 8 act as C items) and the other 16 are fillers of the second slot (8 act as B and 8 act as D). These 32 items and 8 relations are used in such a way across 8 cells of knowledge in the training set that will provide necessary frequency counterbalancing. The cell, as shown in Figure A1B, is divided in two parts, one which contains A:B propositions and one which contains C:D propositions. The two types of propositions have no qualitative differences. They are labeled differently because they serve different roles in the analogy questions we created. In each of the two parts, there are four propositions two of which have a frequency of 3, one with a frequency of 1 and one with a frequency of 5. In summary, a cell has four A:B propositions that have frequencies 3, 1, 3, and 5 and four C:D propositions that again have frequencies 3, 1, 3, and 5. The first A:B proposition is designated to be the source part of the analogy question for that cell. The first C:D proposition is assumed to be the correct response and the other 3 C:D propositions are put as incorrect responses in analogy questions to generate various SFI-type problems.

Since, the first propositions in the two sets are the ones that provide a correct analogy, they share the same relational prototype (but with different instances), while all others have different relations. Analogy tests are obtained by taking the first proposition in the A:B part and the four propositions in the C:D part. The other A:B propositions exist for counterbalancing purposes. Now we need to clarify how counterbalancing occurs. Counterbalancing follows a simple rule. We will try to make all As, Bs, Cs, Ds, and Rs to appear in all possible propositions-rows, so that in total they will all have the same frequency.

Figure A1B shows the general format of a cell. All A:B and C:D propositions in cell *j* use the same *A_j_* and *C_j_*. Thus, all A and C items have the same frequency in the training set (frequency of 12). Within each cell there is one main relation, and six additional ones. The main relation used in cell *j* is relation *R*j. Except for the first A:B proposition and the first C:D proposition that are assigned relation *R_j_*, all other propositions are assigned a sequence of relations that starts from *R_j_*_+1_ (*R*_8_ is followed by *R*_1_). This way each relation appears in all possible row-propositions and provides necessary relational counterbalancing. For example relation *R*_1_ would appear in the first and fourth proposition in cell 1, in the second proposition in cell 8, in the third proposition in cell 7, and so forth, and would not appear at all in cell 2. Finally, each cell has a subset of the 8 B and the 8 D items. This subset is a pseudo-random permutation of 4 integers from 1 to 8. These permutations were constrained. One constraint is that the B (or D) items in a single cell have all to be different. The item *B_i_* cannot appear twice in a cell. Also, each *B_i_* (and *D_i_*) had to appear four times in the entire training set (appear in four cells) appearing in each cell in a different proposition-row. This way all B and D items appear once and only once in all four different row types through the training set. A final constraint is that if a B (and D) and a specific relation R were associated in a given proposition, they should not be associated in a different proposition elsewhere in the training set. That is for controlling for the conditional probability of an item given another item. The number of possible B and D permutation assignments gives us the freedom to create different training sets and test the network in a number of random training environments that obey the same principles, giving us this way more reliable results.

### Relational representations

We also need to clarify our assumptions for relational patterns representations. As we said before relations in the training set come from relational prototypes. For two different relations the prototypes have no overlap at all on their set of active units. But not all occurrences of a prototype are the same. Each relational representation appears in relational instances instead of prototypes. As described above, there are 8 instances of a prototype in the training set. Instances have the same inactive units and have high correlation of active units. An instance is obtained by turning off two specific units of the active units of the prototype. The turned off units are necessarily different across instances. Each prototype has 16 units on, and two of them are turned off in each instance. So, two distinct instances of a prototype have an overlap of 12 active units. Figure A1C shows examples of relational prototypes and instances.

### Network parameters

The A and C pools of the network had 16 units (one for each A and C item in the training set) and the B and D pools had 16 units as well (one for each B and D item). The hidden pools had 110 units and the R pool had 128 units. As noted earlier patterns in the A, B, C, and D pools were localist and in the R pool distributed. Weights were initialized to have random values between −0.25 and 0.25. Activation at each time-step was computed by the logistic function of the net input. We trained 5 networks for 350 epochs with the backpropagation through time algorithm. We used 7 intervals and 4 ticks. For training, input was clamped during the entire processing, while the target error was computed for the last 2 intervals. Training consisted of all three possible input combinations (A:_:B, A:R:_, and _:B:R). We used a learning rate of 0.001 and weight decay of 0.000001. In each training session noise was added in a relational instance so that in expectation one active prototype unit would be turned off in the target and one inactive would be turned on in the target. We used cross-entropy as an error measure. For testing we clamped the A, B, C, patterns for the entire processing while the D1 and D2 patterns were clamped only for 3 intervals. We used the relative echo of the D1 and D2 units as a response decision-criterion.
